# Limited-view signal recovery with frequency-aware denoising diffusion and geometry-integrated masked autoencoders for X-ray acoustic computed tomography

**DOI:** 10.1016/j.pacs.2026.100826

**Published:** 2026-04-10

**Authors:** Jiayuan Peng, Mengyang Lu, Bin Cai, Weigang Hu, Qingli Zhou, Xin Liu

**Affiliations:** a College of Biomedical Engineering, Fudan University, Shanghai, China; b Department of Radiation Oncology, Fudan University Shanghai Cancer Center, Shanghai, China; c Department of Oncology, Shanghai Medical College, Fudan University, Shanghai, China; d Department of Radiation Oncology’s Division of Medical Physics & Engineering, University of Texas Southwestern Medical Center, Dallas, TX, USA; e Department of Information Technology, International Institutes of Medicine, the Fourth Affiliated Hospital of School of Medicine and International School of Medicine, Zhejiang University, Yiwu, China; f State Key Laboratory of Brain Function and Disorders, Fudan University, China

**Keywords:** X-ray acoustic computed tomography (XACT), Limited-view signal recovery, Frequency-aware denoising (FAD), Geometry-integrated masked autoencoders (GIMAE), Inverse reconstruction

## Abstract

X-ray acoustic computed tomography (XACT) suffers from low SNR and limited-view detection when only a limited number of detector channels are available. To address these limitations, we introduce a two-stage limited-view signal recovery framework that integrates frequency-aware denoising (FAD) diffusion with geometry-integrated masked autoencoders (GIMAE). FAD jointly models temporal and spectral noise characteristics to restore clean RF measurements, while GIMAE leverages detector-layout-guided masking to infer missing angular channels and reconstruct full-view RF sinograms from limited-view inputs. The recovered signals are subsequently used for XACT image reconstruction via time-reversal algorithm. Simulation and experimental evaluations using multiple X-ray irradiated patterns demonstrate substantial improvements in reconstruction fidelity, with the proposed method boosting SSIM by 31.1% in simulation and by 26.4% in experiments—closely matching full-view references and outperforming limited-view acquisitions. This framework provides an effective and practical solution for high-quality limited-view XACT imaging under realistic noise-dominated conditions.

## 1 Introduction

X-ray acoustic computed tomography (XACT) has recently gained attention as a thermoacoustic imaging modality capable of mapping energy deposition from ionizing radiation inside attenuating media [1], [2], [3], [4], [5], [6], [7], [8], [9], [10], [11]. When pulsed X-rays are absorbed, rapid thermoelastic expansion generates pressure waves whose temporal signatures encode the underlying spatial dose distribution. These advances underscore the potential of XACT to bridge dose imaging in radiation physics with acoustic sensing, offering hybrid imaging capabilities not achievable with conventional dose measurement in radiotherapy (RT).

This capability directly addresses a long-standing unmet need in RT. Although RT is a cornerstone of cancer management—received by nearly half of all cancer patients [12]—the field continues to lack a reliable method for real-time, in vivo verification of delivered dose. Current practice relies largely on phantom-based measurements or external detectors placed outside the patient [13], [14], which cannot directly resolve internal dose deposition and therefore provide limited feedback during treatment. In principle, XACT offers exactly the missing functionality: a noninvasive mechanism for mapping deposited dose in real time during irradiation.

Despite this promise, practical implementation of XACT under megavoltage (MV) X-ray beams remains challenging. Clinical treatment setups impose strict geometric constraints around the patient and treatment hardware, frequently resulting in limited-angle detector arrangements. Such incomplete angular sampling produces sinograms with missing views, exacerbating the ill-posedness of the inverse problem and leading to blurred structures, intensity non-uniformity, and loss of spatial fidelity in reconstructed XACT images.

Similar challenges occur in CT and MRI, where sparse or limited-angle data acquisition leads to ill-posed inverse problems and degraded image quality [15], [16]. Recently, deep learning models trained on large datasets have shown strong capabilities in predicting missing measurements and reducing artifacts, enabling higher-quality and even real-time reconstruction [15], [16].

Motivated by these advances, we posit that XACT radiofrequency (RF) signals possess sufficient spatial and angular redundancy such that partial-angle measurements may still permit recovery of the full-view sinogram. Specifically, we hypothesize that missing angular RF channels can be inferred by combining acquired limited-view measurements with prior-informed recovery patterns learned from training data. To test this hypothesis, we develop and evaluate a signal-domain limited-to-full view RF recovery framework using both simulation studies and real MV XACT measurements, enabling physically consistent reconstruction under practical limited-view conditions.

Through combined simulation and experimental validation on a medical MV linear accelerator (LINAC), we demonstrate that the proposed framework suppresses band-limited and white noise, restores waveform integrity, recovers missing angular information, and substantially improves the fidelity of reconstructed XACT dose distributions.

## 2 Methods

### 2.1 Overall framework

We propose a two-stage framework for limited-view signal recovery, consisting of a frequency-aware denoising (FAD) diffusion model and a signal recovery network based on geometry-integrated masked autoencoders (GIMAE).

**Fig. 1:**
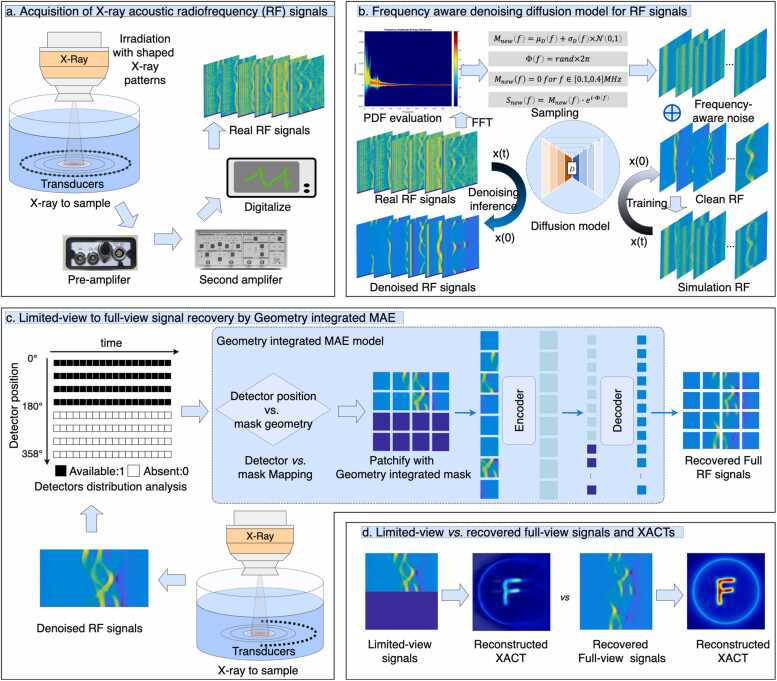
Overview of the proposed frequency-aware denoising and geometry-integrated masked autoencoders for limited-view signal recovery in X-ray Acoustic Tomography (XACT).

As illustrated in Fig. 1, raw RF signals are acquired under shaped X-ray excitation, amplified, and digitized (Fig. 1a). The FAD module first suppresses band-limited and white noise to generate denoised full-view RF signals (Fig. 1b) [17].

These signals are then subsampled according to the acquisition geometry to form limited-view inputs. The GIMAE module reconstructs the missing angular channels to recover complete 360° RF sinograms (Fig. 1c), which are subsequently used for XACT image reconstruction (Fig. 1d).

### 2.2 Signal acquisition

As illustrated in Fig. 1a, X-ray-induced acoustic signals were acquired by irradiating a water tank using a LINAC (Edge, Varian Medical Systems, USA) operating in 10 MV flattening-filter-free (FFF) mode at a dose rate of 2400 MU/min. The 10 MV FFF beam is delivered as microsecond-scale pulses, with a pulse width of approximately 4 μs and a repetition rate of 360 Hz under these settings.

Acoustic pressure waves generated by X-ray energy deposition were detected using a 0.5 MHz immersion ultrasound transducer (V301-SU, Olympus-NDT, USA) positioned 2 cm below the water surface. The detected RF signals were sequentially amplified using a pre-amplifier (CTS-8682A, Shantou Ultrasonic Electronics, China) and a low-noise voltage pre-amplifier (SR560, Stanford Research Systems, USA), and subsequently digitized by an oscilloscope (DSOX-1202A, Agilent Technologies, USA). A photograph of the complete experimental setup is provided in Supplementary Fig. S1.

Because a dedicated ring-array transducer system was not available, multi-angle RF measurements were obtained by rotating the collimator in 2° increments to emulate near-circular angular sampling around the tank as illustrated in Supplementary Fig. S1. This procedure provided 0–350° of angular coverage, with four missing 2° positions (352°, 354°, 356°, and 358°) due to mechanical constraints of the LINAC head and multileaf collimator (MLC) system. As a result, the experimentally acquired RF sinograms consisted of 176 detector angles and 1000 time samples per angle, with a temporal sampling interval of $1\times 10^{-7}\text{s}$. For each irradiation angle, RF measurements were averaged over 8192 X-ray pulses to ensure stable acquisition.

### 2.3 Frequency-aware denoising diffusion model

The first stage of the proposed framework performs frequency-aware denoising [17] to suppress both band-limited noise and white noise in measured RF signals prior to angular subsampling and limit-to-full view recovery (Fig. 1b).

As illustrated in Fig. 1b, the frequency statistics of noise are first characterized using experimentally measured RF signals. Each RF signal is transformed into the frequency domain via fast Fourier transform (FFT). The magnitude spectra are averaged and normalized to obtain a spectral probability density function (PDF), which represents the distribution of noise energy across frequency components.

Based on the estimated PDF, frequency-aware noise is then synthesized in the frequency domain. For each frequency component, the magnitude is sampled from a normal distribution parameterized by the mean and standard deviation derived from the PDF, while the phase is randomly drawn from a uniform distribution in [0,2π]. Frequency components within 0.1–0.4 MHz are suppressed to avoid overlap with useful signal components. The sampled magnitude and phase are combined and transformed back to the time domain using inverse FFT, yielding band-limited noise. Additional stochastic white noise is further added to simulate realistic measurement conditions.

Clean RF signals are generated using k-Wave simulations based on initial pressure distributions derived from non-experimental x-ray beam patterns. The synthesized frequency-aware noise and white noise are added to these clean RF signals to form noisy RF signals, thereby constructing paired noisy and clean RF data for training.

A conditional denoising diffusion model [18] is trained to learn the mapping from noisy RF signals to clean RF signals. During training, Gaussian noise is progressively added to the input, and the network is optimized to predict the injected noise at each diffusion timestep using a mean squared error (MSE) loss.

During inference, the reverse diffusion process is applied to real measured RF signals. Starting from noisy input *x(t)*, the model iteratively removes noise to obtain denoised RF signals *x(0)*, enabling progressive suppression of both structured band-limited noise and unstructured random noise

### 2.4 Limited-view signal recovery by GIMAE

Building upon the standard masked autoencoders framework [19], GIMAE incorporates detector layout into the geometry-aware masking strategy by encoding the spatial locations of missing detector channels as illustrated in Fig. 1c. This geometry-informed masking allows the model to reconstruct limited-view measurements in a manner that is consistent with the physical acquisition layout, rather than treating all missing signals as generic random gaps.

Formally, let

$$x\in R^{N\times T}$$

denote the full set of acoustic signals from *N* detectors over *T* time samples. In our implementation, each RF signal matrix was resampled to 256 × 256 prior to patch embedding, following the ViT-MAE input convention. Limited-view acquisition is modeled as

$$x_{s}=M⊙x$$

where $M\in {\left\{0,1\right\}}^{N}$ is a geometry-integrated mask determined by detector availability (*M*_*i*_=1 if detector *i* is present, 0 otherwise). The encoder $E\left(\cdot \right)$ maps masked signals into a latent representation, while the decoder $D\left(\cdot \right)\;$reconstructs the missing channels:

$$\hat{x}=D\left(E\left(x_{s},M\right)\right)$$

BecauseMis constructed from the actual detector topology, the reconstruction $\hat{x}$ is physically constrained to match the acquisition geometry, representing an extension of MAE toward geometry-aware **limited-view signal recovery**.

The model was trained using a masked mean squared error (MSE) loss computed only on the masked elements:

$$L_{\mathrm{GIMAE}}=\frac{1}{{\left\|1-M\right\|}_{0}}\sum_{i,t}\left(1-M_{i}\right)\;{\left\|x_{i,t}-{\hat{x}}_{i,t}\right\|}^{2}$$

Here, M denotes the geometry-integrated mask;$1-M_{i}$ indicates the masked positions; the loss is computed only over the masked entries, consistent with ViT-MAE; and the normalization term ${\left\|1-M\right\|}_{0}$ represents the total number of masked elements.

In practice, the 256 × 256 input x was first divided into non-overlapping patches, each projected into patch embeddings with positional encodings. Using the mapping between detector layout and the patch grid, patches corresponding to missing detector channels were masked to produce the limited-view representation$\;x_{s}$. The ViT encoder then operated exclusively on the visible (unmasked) tokens, producing latent representations. The decoder subsequently fused these latent tokens with learned mask tokens to reconstruct both visible and masked patch embeddings. Finally, the reconstructed embeddings were projected back and reshaped into the N×T detector-time domain, yielding the recovered full-view RF signal$\hat{\;x}$.

### 2.5 XACT Image Reconstruction

XACT image reconstruction was performed using a two-dimensional time-reversal algorithm implemented in the k-Wave MATLAB toolbox (MathWorks, Natick, MA, USA).

Recovered RF signals were applied as the boundary condition for the time-reversal algorithm, which propagated the wavefield backward in time to estimate the initial pressure distribution. The resulting pressure map served as the reconstructed XACT image for subsequent analysis.

### 2.6 Simulation study

A simulation study was conducted prior to the physical experiments to evaluate limited-view signal recovery. Two complex X-ray patterns (peace dove and leaf) were generated using a dynamic sliding-window MLC sequence and simulated in the Varian Eclipse treatment planning system (TPS, Varian Medical Systems, USA) with a 10 MV beam to obtain dose distributions in water.

The dose maps were converted into initial acoustic pressure fields and propagated using k-Wave under both homogeneous and heterogeneous media, mimicking water-equivalent tissue and more complex acoustically heterogeneous biological samples, respectively. The homogeneous simulations were performed using a uniform water background, while the heterogeneous simulations shared the same simulation setup and parameters, except that three acoustic inclusions with different sound speeds and densities were embedded in the water background. Full-view RF sinograms with 180 detector views (2° spacing) were generated. Detailed simulation parameters, including the heterogeneous acoustic medium configuration and sensor geometry used in the k-Wave simulations, are provided in Supplementary Section S2.

For GIMAE training, additional synthetic pressure patterns were created using alphabet and geometric shapes with deformation-based augmentation. For the homogeneous and heterogeneous configurations, forward simulations were performed separately, each producing paired half-arc limited-view and full-view RF signals, with 660 training samples and 85 validation samples per configuration.

After training, GIMAE was evaluated on the peace dove and leaf patterns by recovering full-view RF signals from half-arc inputs. The recovered RF signals were reconstructed into XACT images using the time-reversal method described in 2.5 and the same k-Wave parameters (Supplementary Section S2), enabling comparison among full-view, limited-view, and recovered full-view reconstructions.

### 2.7 Experimental study

To generate diverse acoustic sources, six X-ray beam patterns were designed in the TPS and delivered using the LINAC with MLC beam shaping:

(1) three English letters (F, U, and N),

(2) a pattern consisting of three isolated micro-lesion–like spots,

(3) a paired geometry pattern composed of two adjacent but non-connected shapes, and

(4) a dual-line X-ray grating pattern.

These patterns were irradiated onto the water tank, producing distinct initial pressure fields and the corresponding near “full-view” RF signal sets (referred to as “full-view” or “full-arc” hereafter for clarity) used for denoising and limited-to-full-view recovery evaluation. An example of the X-ray irradiation for the letter “F” pattern is shown in Supplementary Fig. S1.

The full-view RF signals, acquired as described in 2.2, were first processed using the FAD method (2.3). The trained FAD model was then applied to the experimentally measured RF signals to obtain denoised full-view RF sinograms for subsequent subsampling and recovery.

Limited-view (half-arc) sinograms were generated by subsampling the denoised RF signals to 90 × 1000 (180° coverage at 2° spacing). The detector geometry was encoded into the mask used by GIMAE, and the limited-view inputs were processed to recover full 180 × 1000 RF sinograms (Fig. 1c; 2.4). The recovered signals were subsequently reconstructed into XACT images using the time-reversal method described in 2.5.

To train the GIMAE network, paired limited-view and full-view datasets were constructed from synthetic clean RF signals with small Gaussian perturbations to mimic residual noise after FAD denoising, resulting in 2342 training samples and 624 validation samples.

### 2.8 Training and comparative models

#### 2.8.1 FAD model

A total of 6456 paired noisy-clean RF samples were used for training. The original RF signals were resized to 256 × 256 and used as model inputs. The FAD model was trained for 880,000 iterations with a batch size of 8 using the AdamW optimizer (learning rate: 1 ×10⁻⁴). The training objective was the mean squared error (MSE) between the predicted and ground-truth noise at each diffusion timestep. No validation set was employed; convergence was assessed based on the stability of the training loss (Supplementary Fig. S3). Training required approximately 14 days. During inference, the 1000-step diffusion sampling procedure was adopted, with an average runtime of approximately 45 s per sample.

#### 2.8.2 GIMAE model

A total of 2342 training samples and 624 validation samples were used for GIMAE training. The input RF signals were resized to 256 × 256 prior to patch embedding. The GIMAE model was implemented using a ViT-MAE architecture with a 16 × 16 patch size, an encoder dimension of 2048 with 8 transformer layers and 16 attention heads, and a 12-layer decoder with 2048-dimensional embeddings. Training was performed for 5000 epochs using the AdamW optimizer (learning rate: 1 ×10⁻⁴, weight decay: 0.05) with a cosine learning-rate schedule and 1% warm-up. The training objective followed the masked mean squared error (MSE) loss defined in 2.4. Convergence was monitored based on the validation loss, and the model achieving the lowest validation loss was selected for testing (Supplementary Fig. S3). Training required approximately 4.5–5 days. During inference, the average runtime was approximately 0.034 s per sample.

#### 2.8.3 Comparative models

Three representative image-domain recovery models were implemented for comparison, including FD-UNet [20], a generative adversarial network (GAN) [21], and a diffusion model (DM) [18]. All baseline models were trained to recover high-quality XACT images from low-quality reconstructions obtained under limited-view conditions. To ensure a fair comparison, identical training and validation splits were adopted across all methods. The training hyperparameters for each network were independently optimized to achieve stable convergence. The number of training epochs differed according to model characteristics: FD-UNet and GAN were trained for 500 epochs, whereas the diffusion model was trained for 3000 epochs.

#### 2.8.4 Hardware and software environment

All training experiments were conducted on a workstation equipped with an NVIDIA RTX 6000 Ada (48 GB) GPU, an AMD Threadripper PRO 7945WX CPU, and 512 GB of RAM, running Ubuntu 20.04, PyTorch 2.0.1, and CUDA 11.7.

#### 2.8.5 Statistical analysis

Quantitative results are reported as mean ± standard deviation unless otherwise stated. Statistical significance for paired comparisons was assessed using the Wilcoxon signed-rank test, with *p* < 0.05 considered statistically significant.

## 3 Results

### 3.1 Simulation study

**Fig. 2:**
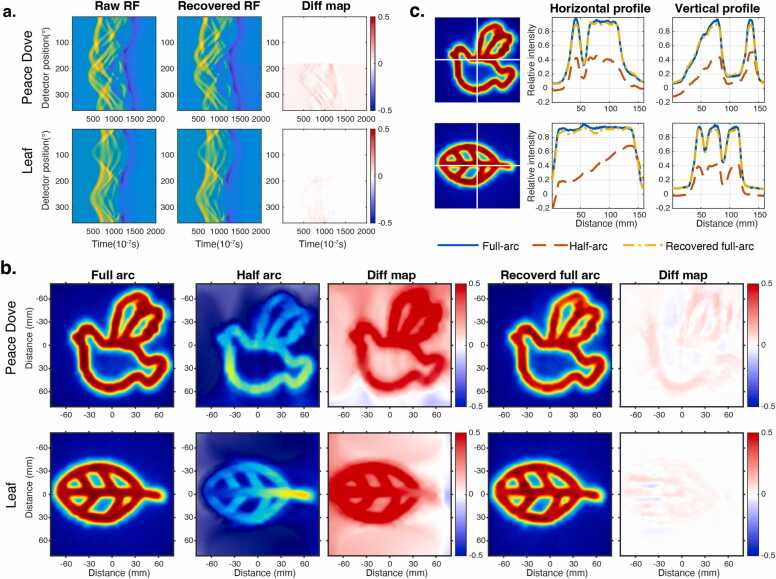
(a) Comparison of raw, recovered, and their difference maps. (b) XACT reconstructions from full-arc, half-arc, and recovered full-arc RF inputs (images are rotated for visual consistency). (c) Horizontal and vertical line-profile comparisons.

Fig. 2 summarizes the simulation results obtained in the homogeneous (water-equivalent) medium.

As shown in Fig. 2a, the proposed model successfully reconstructs full-arc RF measurements from half-arc inputs. The recovered waveforms exhibit high temporal agreement with the ground truth, including consistent amplitude evolution and phase continuity. Normalized residual maps reveal only small, edge-localized deviations (typically within ±0.1–0.15), indicating high-fidelity signal recovery. Quantitatively, the recovered RF signals achieved PSNR values of 30.673 dB (Peace Dove) and 34.057 dB (Leaf), together with SSIM of 0.956 and 0.971, computed over the recovered angular region.

Fig. 2b compares XACT reconstructions from full-arc, half-arc, and recovered full-arc RF inputs. For improved visualization and consistent layout, the reconstructed images were post-processed with simple rigid in-plane rotations; this operation does not alter the reconstruction content or quantitative comparisons. As expected, half-arc reconstructions suffer from blurring and directional-dependent intensity non-uniformity due to angular incompleteness. In contrast, reconstructions based on the recovered full-arc RF signals substantially suppress these artifacts and closely reproduce the full-view reference. This improvement is further confirmed by the horizontal and vertical line profiles in Fig. 2c, where the recovered profiles closely follow the ground-truth curves, whereas half-arc inputs exhibit pronounced amplitude loss and intensity mismatch.

Quantitative results summarized in Table 1 consistently demonstrate significant improvements in SSIM, PSNR of the reconstructed XACT images for both patterns, confirming that the proposed limited-view signal recovery effectively mitigates angular incompleteness and restores high-quality XACT reconstructions.

**Table 1:** Quantitative Evaluation of XACT Reconstructions (SSIM, PSNR).

Pattern	Method	SSIM	PSNR (dB)
Peace dove	Recovered Full-Arc	**0.989**	**32.013**
	Half-Arc	0.738	11.875
Leaf	Recovered Full-Arc	**0.991**	**36.595**
	Half-Arc	0.772	12.110

Additional simulation results in heterogeneous media are provided in Supplementary Fig. S5 and Table S1.

### 3.2 Experimental study

#### 3.2.1 Real signal denoising

**Fig. 3:**
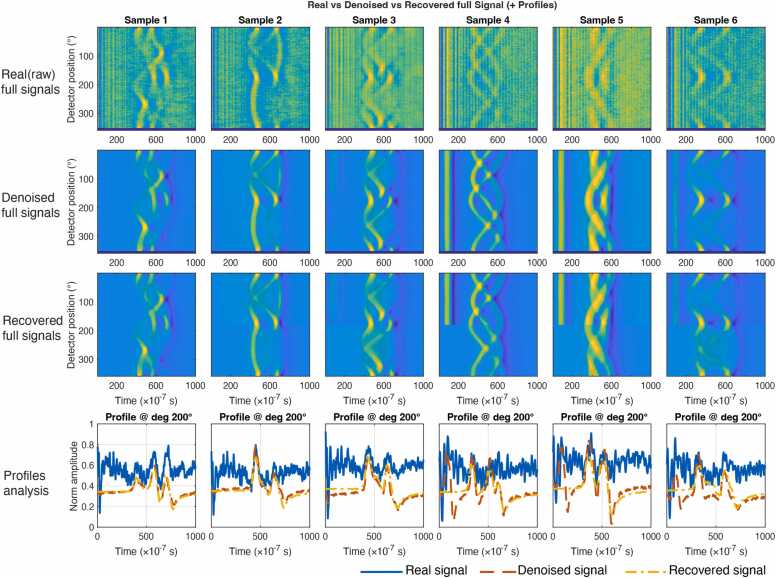
Real, denoised, and recovered full-arc RF signals for six samples. Rows show raw noisy measurements, denoised signals, and recovered full-arc signals from half-arc inputs. The bottom row shows temporal profiles extracted at detector position 200° for real, denoised, and recovered signals.

Fig. 3 compares raw full-arc RF signals (first row) with their denoised counterparts (second row) for six samples. Due to four missing angular views, the raw sinograms contain zero-padded bands and strong background fluctuations that obscure the underlying acoustic structure. After applying FAD denoising, the oscillatory patterns become substantially clearer with reduced noise while preserving high-frequency waveform details.

For Samples 4–6, a weak stripe-like artifact remains near the beginning of the time axis, originating from residual start-up pulse interference of the medical LINAC. This interference is localized around time zero and is well separated from the main effective signal region; therefore, it does not affect subsequent XACT reconstruction.

Table 2 shows that the average SNR increased from 0.03 ± 0.22 dB (raw) to 3.07 ± 0.56 dB after denoising (*p* < 0.05), confirming strong and consistent noise suppression.

**Table 2:** ROI-SNR (dB) of real full signals, denoised full signals, and recovered full signals from denoised limited-view inputs.

Sample	Raw signals (dB)	Denoised signals (dB)	Recover signals (dB)
1	0.34	2.84	3.03
2	-0.25	2.4	2.67
3	0.19	4.09	3.96
4	-0.03	2.94	3.37
5	-0.15	3.17	3.57
6	0.07	2.95	3.24
Average	0.03 ± 0.22	3.07 ± 0.56	3.31 ± 0.44

#### 3.2.2 Limited-to-full view signal recovery

Fig. 3 further compares denoised full-arc signals (second row) with recovered full-arc signals (third row) from half-arc denoised inputs. Visually, the recovered signals closely resemble the fully sampled denoised signals in both global waveform structure and local oscillation patterns. Despite being generated from limited-view inputs, the recovered signals preserve key signal features, including the double-helix-like structure and periodicity. This qualitative consistency indicates that the proposed model can infer missing angular information and restore full-view signal distributions.

Quantitatively (Table 2), the recovered signals achieved an average ROI-SNR of 3.31 ± 0.44 dB, which is statistically comparable to that of the denoised full-arc signals (3.07 ± 0.56 dB; *p* = 0.0625). We note that the recovered full-view signals exhibit slightly higher SNR than the directly denoised full-arc signals, a phenomenon that is further discussed in Section 4.

#### 3.2.3 XACT reconstruction analysis

**Fig. 4:**
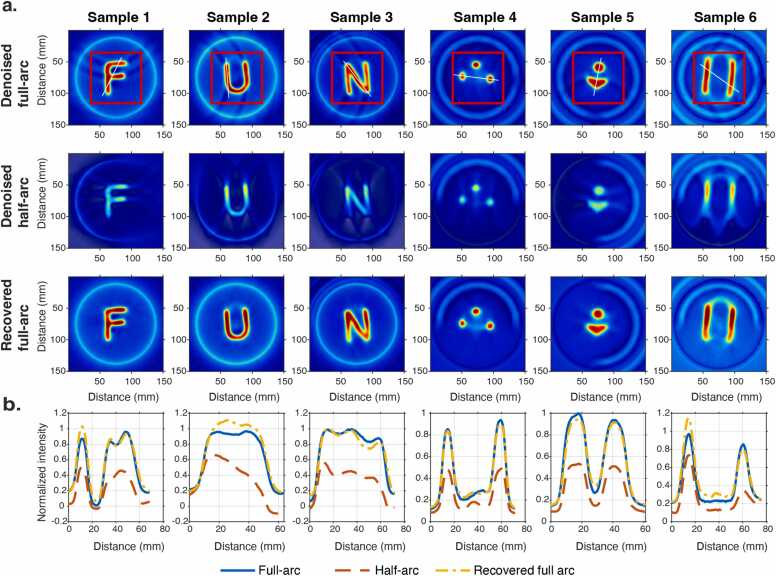
XACT reconstructions and intensity profiles from denoised full-arc, denoised half-arc, and recovered full-arc signals. (a) Reconstructed images for six samples (images are rotated for visual consistency). (b) Corresponding line-profile comparisons extracted along the white lines indicated in the first row.

Fig. 4a shows representative XACT reconstructions from denoised full-arc, denoised half-arc, and recovered full-arc RF signals. For visualization consistency, simple rigid in-plane transformations were applied during figure preparation; these operations affect only image orientation and do not alter reconstruction values or quantitative analysis.

Because the denoised “full-arc” measurements still contain four missing angular views, slight streaks remain visible in their reconstructions (first row). Half-arc reconstructions exhibit much stronger artifacts, including reduced signal intensity, spatial non-uniformity, and blurred edges (second row). In addition, the residual start-up noise observed in the denoised RF signals of Samples 4–6 (Fig. 3) can propagate into the reconstructed XACT images (Fig. 4), leading to more evident peripheral ring-like/half-ring-like artifacts, however, this interference is mainly localized at the periphery and does not overlap with the main imaging region.

In contrast, reconstructions based on the recovered full-arc signals substantially suppress limited-view artifacts and restore structural fidelity and contrast, closely matching the full-arc reference. Quantitatively, compared with denoised half-arc reconstructions, recovered full-arc results show marked improvements in both SSIM (0.759 ± 0.090 to 0.959 ± 0.008) and PSNR (15.874 ± 2.847 dB to 27.464 ± 2.070 dB) (*p* < 0.05), computed within the effective image region indicated by the red boxes in Fig. 4a, demonstrating effective mitigation of limited-view degradation.

Fig. 4b further confirms this through one-dimensional intensity profiles: half-arc inputs show reduced amplitude loss and intensity mismatch, whereas recovered profiles align closely with the full-arc reference, indicating accurate restoration of structural details.

#### 3.2.4 Recovery performance under different detector layouts

**Fig. 5:**
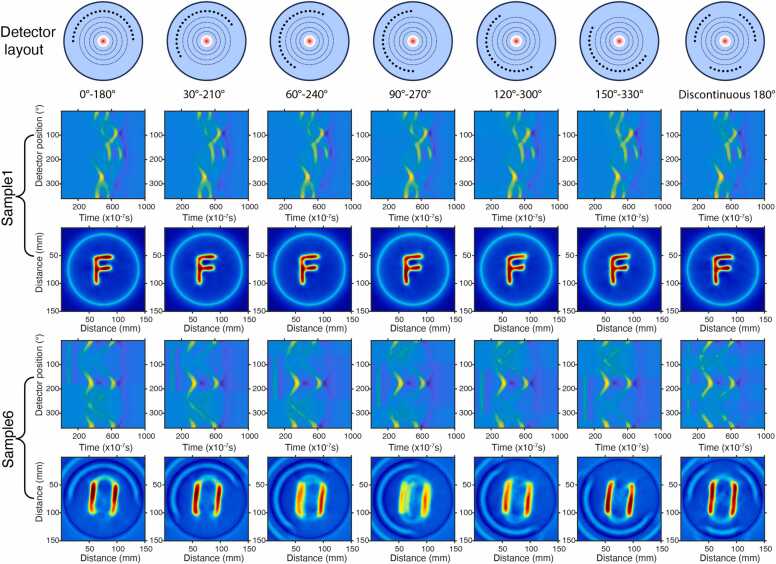
Recovery performance under different detector layouts. Top: angular-gap configurations; middle: recovered RF sinograms; bottom: corresponding XACT reconstructions.

While 3.2.2, 3.2.3 examined limited-to-full recovery under a single detector layout (0°–180°), Fig. 5 evaluates the model’s robustness by applying multiple alternative detector layouts to the same X-ray pattern.

Quantitative analysis in Table 3 shows that the recovery performance remains generally stable across different layouts. Using the 0°–180° configuration as the reference, most alternative layouts do not exhibit statistically significant differences in either SSIM or PSNR (*p* > 0.05). Two rotated layouts (60°–240° and 120°–300°) show statistically significant but modest reductions in both metrics (*p* < 0.05), while the absolute performance degradation remains limited.

**Table 3:** Quantitative comparison of XACT reconstruction performance after RF signal recovery under different detector layouts.

Detector layout	SSIM	PSNR (dB)
0°–180°	0.959 ± 0.008	27.464 ± 2.070
30°–210°	0.948 ± 0.011	26.024 ± 1.578
60°–240°	0.919 ± 0.048*	24.128 ± 3.339*
90°–270°	0.905 ± 0.063	23.220 ± 3.508
120°–300°	0.922 ± 0.040*	24.098 ± 2.391*
150°–330°	0.946 ± 0.015	26.123 ± 1.868
Discontinuous 180°	0.963 ± 0.009	29.16 ± 2.682

* Statistically significant difference compared with the 0°–180° detector layout.

In addition to the rotated continuous half-arc layouts, we further evaluated a discontinuous 180° configuration composed of three alternating 60° sectors, as shown in the last column of Fig. 5. Compared with the continuous 0°–180° reference, this layout does not exhibit statistically significant differences in SSIM or PSNR (*p* > 0.05). The reconstruction quality remains consistently high, further confirming that the proposed method achieves stable performance across diverse detector layouts.

#### 3.2.5 Comparative performance under half-arc and quarter-arc limited-view conditions

We further conduct a comprehensive comparison between the proposed framework and representative image-domain recovery methods under both half-arc and quarter-arc limited-view conditions.

**Fig. 6:**
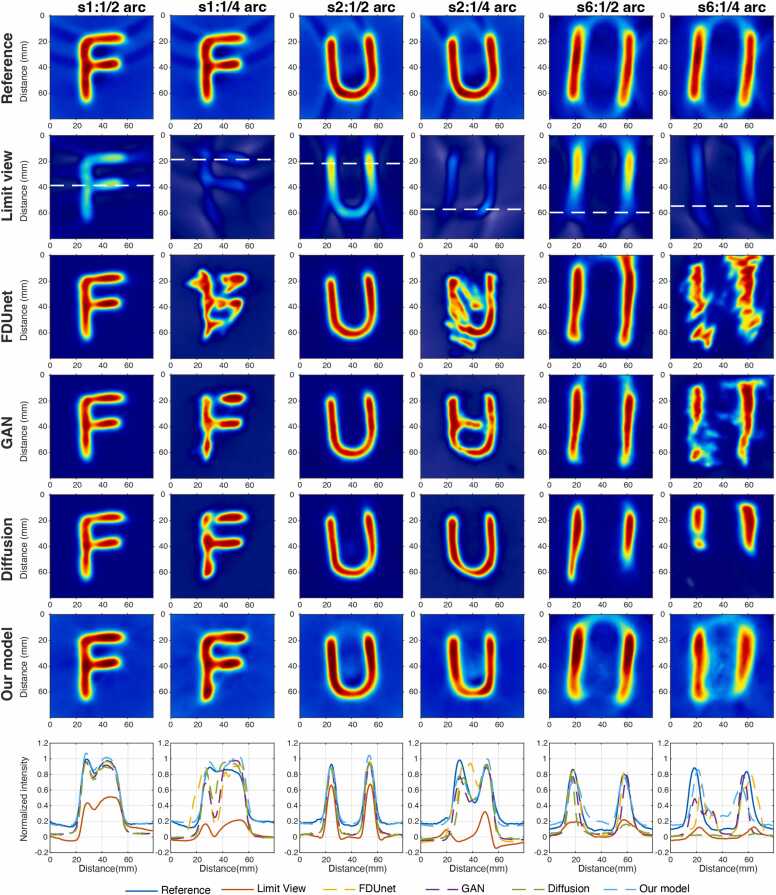
Comparison of XACT reconstructions under half-arc and quarter-arc limited-view conditions using different recovery methods. The bottom row shows corresponding normalized line profiles (white dashed lines) for quantitative comparison across methods.

Fig. 6 and Table 4 summarize the quantitative and qualitative results. Under half-arc sampling, all learning-based recovery methods improve upon the limit-view baseline, indicating effective compensation of moderate angular incompleteness. Among all compared approaches, the proposed GIMAE achieves the highest reconstruction accuracy, with an SSIM of 0.959 ± 0.008 and a PSNR of 27.464 ± 2.070 dB, substantially outperforming FDUNet, GAN, and diffusion-based models (*p* < 0.05).

**Table4:** Quantitative comparison (SSIM and PSNR) of XACT reconstructions under half-arc and quarter-arc limited-view conditions using different recovery methods.

	1/2 arc recovered		1/4 arc recovered	
	SSIM	PSNR (dB)	SSIM	PSNR (dB)
Limit-view	0.759 ± 0.090	15.874 ± 2.847	0.626 ± 0.090	12.506 ± 2.539
FDUNet	0.848 ± 0.063	20.928 ± 3.274	0.675 ± 0.100	16.024 ± 2.621
GAN	0.855 ± 0.071	21.441 ± 3.950	0.768 ± 0.080	18.127 ± 3.584
Diffusion	0.843 ± 0.077	21.233 ± 4.895	0.784 ± 0.074	17.677 ± 3.763
GIMAE(Our)	**0.959 ± 0.008**	**27.464 ± 2.070**	**0.889 ± 0.072**	**22.139 ± 3.052**

As the angular coverage is further reduced to quarter-arc, reconstruction quality degrades for all methods, reflecting the increased extent of information loss. Despite this, GIMAE consistently maintains superior performance, achieving an SSIM of 0.889 ± 0.072 and a PSNR of 22.139 ± 3.052 dB, significantly higher than all competing methods (*p* < 0.05).

## 4 Discussion

In this study, we introduced a signal-domain limited-view signal recovery framework that combines frequency-aware denoising with geometry-integrated masked autoencoders. The FAD module effectively suppresses white and band-limited noise while preserving spectral structures critical for downstream recovery. Based on these denoised measurements, the GIMAE module incorporates signal-domain priors and detector layout to infer missing RF channels in a physically consistent manner.

Both simulation and experimental results demonstrate that our method enables accurate recovery of missing angular information under limited-view conditions. The combined FAD-GIMAE pipeline markedly improves RF completeness and fidelity, yielding XACT reconstructions that closely approach fully sampled quality despite limited-angle acquisition constraints.

It is essential to distinguish this study from prior single-view XACT research. For example, Wang et al [11]. focused on structural imaging, utilizing nanosecond-scale X-ray pulses to generate stronger thermoacoustic signals from targets with high intrinsic spatial absorption contrast. In contrast, our work addresses dose deposition mapping in homogeneous, water-equivalent media. This task is fundamentally more challenging as it involves detecting ultra-weak signals generated by microsecond-scale megavoltage (MV) pulses in a regime with no intrinsic absorption boundaries. Consequently, while prior studies imaged the target's structure, our framework is designed to reconstruct the X-ray beam’s own shape and dose distribution under low signal-to-noise ratio (SNR) conditions.

The effectiveness of the proposed framework relies on two key design choices. First, frequency-aware denoising explicitly models experimentally measured MV noise characteristics, enabling robust noise suppression without sacrificing high-frequency components that are critical for angular recovery. Second, geometry-integrated masking embeds detector layout directly into the learning process, allowing the model to learn physically meaningful angular dependencies rather than treating missing views as independent gaps. This contrasts with MAE-based medical imaging studies where masking is typically random or grid-based in the image domain and primarily serves representation learning or pretraining purposes [22], [23].

We note that recovered full-view RF signals occasionally exhibit slightly higher SNR than directly denoised full-view measurements. This effect reflects the implicit regularization introduced by the geometry-consistent recovery network, which can suppress residual structured noise remaining after denoising. Similar behavior has been reported in related XACT denoising literature. For instance, Jiang et al. observed that deep learning models trained with high-frame-averaged references may yield outputs with slightly higher SNR than the references themselves, an effect commonly attributed to implicit regularization [24].

We note that residual peripheral artifacts observed in some half-arc reconstructions (Fig. 4a, Samples 4–6) are primarily attributable to transient LINAC start-up interference near time zero in the RF signals (Fig. 3), which is consistent with the known susceptibility of piezoelectric ultrasound transducers to electromagnetic interference [25].

This study has several limitations. While heterogeneous acoustic conditions were explored in simulations, the experimental dataset remains limited in diversity and was acquired in a homogeneous, tissue-equivalent water medium. In addition, real-time implementation was not investigated and will require further optimization. Nevertheless, the proposed framework provides a robust and generalizable strategy for limited-to-full view recovery in XACT and may benefit other limited-view acoustic and tomographic imaging modalities.

## 5 Conclusion

We proposed a two-stage framework that integrates frequency-aware denoising with geometry-informed masked autoencoders to recover full-angle RF measurements from limited-view XACT acquisitions. Both simulation and experimental results demonstrate that the method restores physically consistent waveforms and substantially mitigates limited-angle artifacts, yielding XACT images close to fully view reconstructions. These findings indicate that the framework offers a practical and effective pathway toward improving in vivo dose imaging in radiotherapy.

## CRediT authorship contribution statement

**Jiayuan Peng:** Writing – review & editing, Writing – original draft, Visualization, Validation, Supervision, Methodology, Investigation, Formal analysis, Conceptualization. **Xin Liu:** Validation, Supervision, Project administration, Methodology, Funding acquisition, Conceptualization. **Qingli Zhou:** Visualization, Supervision, Conceptualization. **Weigang Hu:** Visualization, Supervision, Resources, Conceptualization. **Bin Cai:** Methodology, Conceptualization. **Mengyang Lu:** Visualization, Validation, Formal analysis, Conceptualization.

## Funding

This work was supported in part by research grants from the 10.13039/501100001809National Natural Science Foundation of China (12274092), by the 10.13039/501100012166National Key Research and Development Program of China (2023YFC2410903), by the International Science and Technology Cooperation Program of Shanghai (24490710400), and by the AI for Science Foundation of Fudan University (FudanX24AI016).

## Declaration of Competing Interest

The authors declare that they have no known competing financial interests or personal relationships that could have appeared to influence the work reported in this paper.

## Data Availability

Data will be made available on request.
