# Supplementary material for: Limited-view signal recovery with frequency-aware denoising diffusion and geometry-integrated masked autoencoders for X-ray acoustic computed tomography
Source: Photoacoustics. 2026 Apr 10;49:100826. doi: 10.1016/j.pacs.2026.100826 (PMC13103586; doi:10.1016/j.pacs.2026.100826)
Supplement: Supplementary file 1 — Supplementary material [file mmc1.docx]

**Limited-View Signal Recovery with Frequency-Aware Denoising Diffusion and Geometry-Integrated Masked Autoencoders for X-ray Acoustic Tomography**

Jiayuan Peng^a,b,c^, Mengyang Lu^a^, Bin Cai^d^, Weigang Hu^b,c,*^, Qingli Zhou^e,*^, Xin Liu^a,f,*^

^a^ College of Biomedical Engineering, Fudan University, Shanghai, China

^b^ Department of Radiation Oncology, Fudan University Shanghai Cancer Center, Shanghai, China

^c^ Department of Oncology, Shanghai Medical College, Fudan University, Shanghai, China

﻿^d^ Department of Radiation Oncology’s Division of Medical Physics & Engineering, University of Texas Southwestern Medical Center, Dallas, TX, USA

^e^ Department of Information Technology, International Institutes of Medicine, the Fourth Affiliated Hospital of School of Medicine and International School of Medicine, Zhejiang University, Yiwu, China

^f^ State Key Laboratory of Brain Function and Disorders, Fudan University

**^*^Corresponding authors:**

Weigang hu: [jackhuwg@gmail.com](mailto:jackhuwg@gmail.com)

Qingli Zhou: [zhouql@zju.edu.cn](mailto:zhouql@zju.edu.cn)

Xin Liu: [xin_liu@fudan.edu.cn](mailto:xin_liu@fudan.edu.cn)

**S1: Complete experimental setup for signal acquisition.**


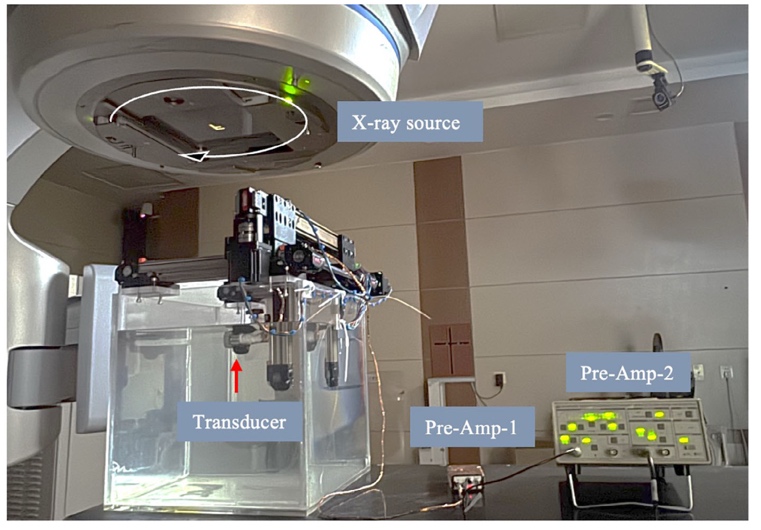


Fig.S1. Photograph of the experimental setup for MV XACT measurements, including the Varian Edge LINAC head, water tank phantom, ultrasound transducer, and signal acquisition chain. In this example, the X-ray beam was shaped into an “F” pattern by the multileaf collimator (MLC) inside the LINAC head and irradiated into the water tank. By rotating the LINAC X-ray source head (indicated by white arrows), while keeping the ultrasound transducer fixed in position, RF signals were acquired over a full angular range, enabling full-view RF measurements. The detected signals were first amplified by a low-noise preamplifier (Pre-Amp-1) and subsequently conditioned by a secondary amplifier (Pre-Amp-2) before digitization.

**S2. Heterogeneous medium configuration and sensor geometry in 2D k-Wave simulations**

To evaluate the robustness of the proposed framework under heterogeneous acoustic propagation, we performed additional 2D k-Wave simulations using a spatially varying medium. The computational domain was discretized on a $300 \times300$ Cartesian grid with isotropic spacing $dx=dy=1\text{mm}$ (physical field-of-view$\approx300\text{mm}\times300\text{mm}$). The temporal grid was manually specified with $dt=1\times{10}^{-7}\text{s}$ and $N_{t}=2000$.

The background medium was water-like with uniform sound speed $c_{0}=1500\text{m}\text{/}\text{s}$ and density $\rho_{0}=1000 \text{kg}\text{/}\text{m}^{\text{3}}$. To introduce controlled heterogeneity, three circular inclusions were embedded at fixed relative positions in the grid. Their radius were $r_{1}=12\text{mm}$, $r_{2}=15\text{mm}$, and $r_{3}=10\text{mm}$.

Each inclusion was assigned distinct acoustic properties to emulate heterogeneous regions (example values):

Inclusion 1: $c=1600 \text{m}\text{/}\text{s}$, $\rho=1100 \text{kg}\text{/}\text{m}^{\text{3}}$

Inclusion 2: $c=1450 \text{m}\text{/}\text{s}$, $\rho=950 \text{kg}\text{/}\text{m}^{\text{3}}$

Inclusion 3: $c=1700 \text{m}\text{/}\text{s}$, $\rho=1200 \text{kg}\text{/}\text{m}^{\text{3}}$

For data acquisition, a centered circular sensor array was used with radius $R=0.14\text{m}$ (140 mm). The sensor consisted of 180 sampling points uniformly distributed over the angular range $0^{\circ}$to ${358}^{\circ}$. Figure S2 visualizes the heterogeneous sound speed and density maps with the overlaid circular sensor geometry.

Fig.S2. Heterogeneous medium maps and circular sensor configuration used in 2D k-Wave simulations. Left: spatial distribution of sound speed $c\left( r \right)$. Right: spatial distribution of density $\rho\left( r \right)$. The red dots indicate the circular sensor positions ($R=140 \text{mm}$, 180 points, $0^{\circ}-{358}^{\circ}$).

**S3: Training loss curves of the two-stage framework.**


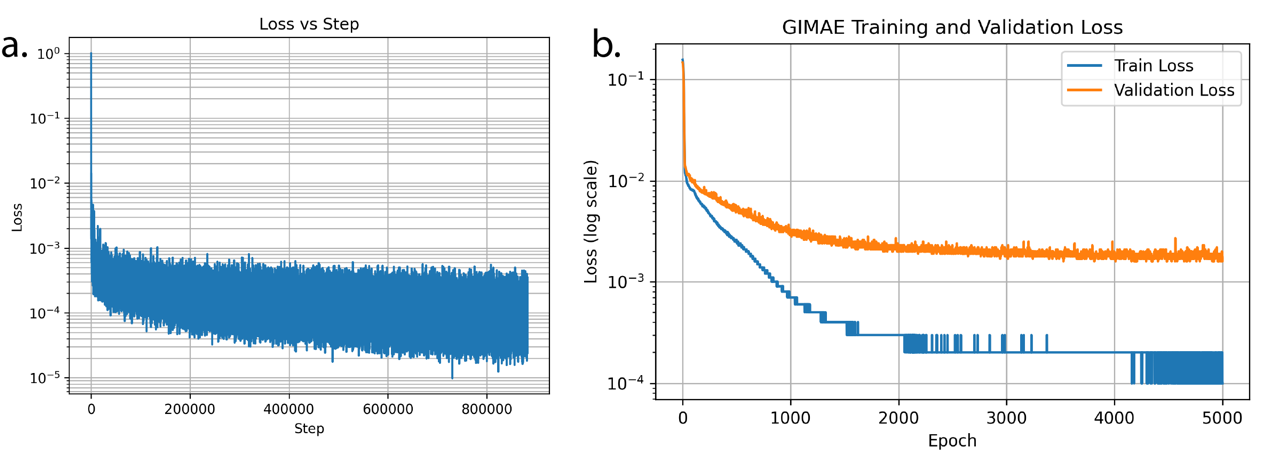


Fig.S3. Training loss curves of the two-stage framework. (a) Training loss of the frequency-aware denoising (FAD) diffusion model over 880,000 iterations. The loss corresponds to the mean squared error (MSE) between the predicted noise and the ground-truth noise at each diffusion timestep. No validation set was employed; convergence was assessed based on the stability of the training loss. (b) Training and validation loss curves of the geometry-integrated masked autoencoder (GIMAE) model over 5,000 epochs, optimized using masked MSE loss.

**S4: Simulation study in heterogeneous media**


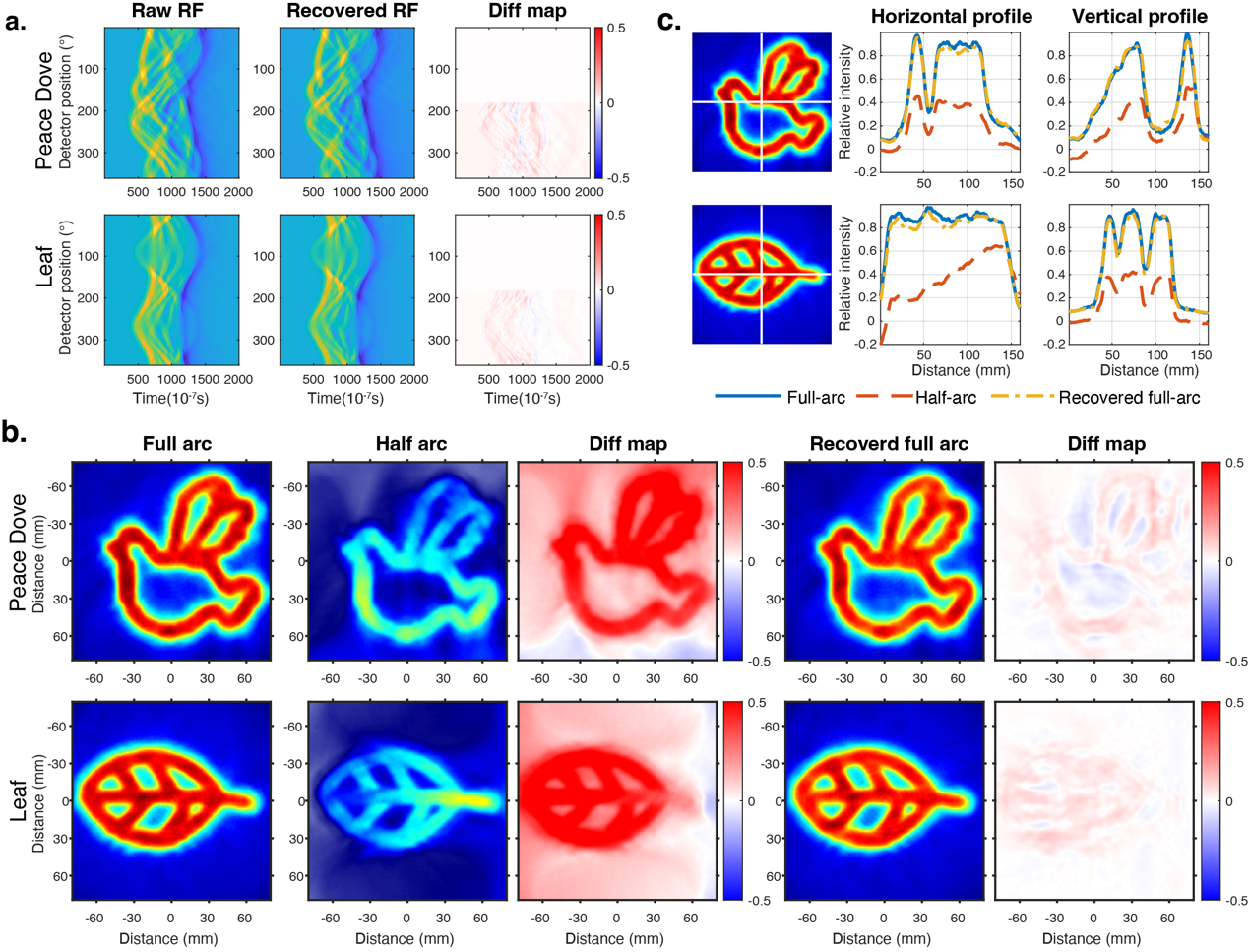


Fig.S4: Performance evaluation under heterogeneous acoustic media. (a) Comparison of raw, recovered, and their difference maps in a heterogeneous medium. (b) XACT reconstructions from full-arc, half-arc, and recovered full-arc RF inputs under heterogeneous propagation conditions. (c) Horizontal and vertical line-profile comparisons.

Table S1 Quantitative Evaluation of XACT Reconstructions (SSIM, PSNR)

| Pattern | Method | SSIM | PSNR (dB) |
| --- | --- | --- | --- |
| Peace dove | Recovered Full-Arc | **0.982** | **32.544** |
|  | Half-Arc | 0.724 | 12.057 |
| Leaf | Recovered Full-Arc | **0.988** | **34.165** |
|  | Half-Arc | 0.724 | 12.383 |
